# Blocking channels to metastasis: targeting sodium transport in breast cancer

**DOI:** 10.1186/s13058-023-01741-1

**Published:** 2023-11-10

**Authors:** William J. Brackenbury, Carlo Palmieri

**Affiliations:** 1 Department of Biology, University of York, Wentworth Way, Heslington, York YO10 5DD UK; 2 York Biomedical Research Institute, University of York, Wentworth Way, Heslington, York YO10 5DD UK; 3 Institute of Systems, Molecular and Integrative Biology, Molecular and Clinical Cancer Medicine, University of Liverpool, Liverpool, UK; 4 The Clatterbridge Cancer Centre NHS Foundation Trust, Liverpool, UK

**Keywords:** Calcium, Potassium, Sodium, Ion channels, Metastasis, Anticonvulsants, Antiarrhythmics

## Abstract

The development of therapies that can suppress invasion and prevent metastasis, ‘anti-metastatic drugs’, is an important area of unmet therapeutic need. The new results of a recent open-label, multicentre randomised trial published in *J Clin Oncol* showed a significant disease-free survival (DFS) benefit for breast cancer patients receiving presurgical, peritumoral injection of lidocaine, an amide local anaesthetic, which blocks voltage-gated sodium channels (VGSCs). VGSCs are expressed on electrically excitable cells, including neurons and cardiomyocytes, where they sustain rapid membrane depolarisation during action potential firing. As a result of this key biophysical function, VGSCs are important drug targets for excitability-related disorders, including epilepsy, neuropathic pain, affective disorders and cardiac arrhythmia. A growing body of preclinical evidence highlights VGSCs as key protagonists in regulating altered sodium influx in breast cancer cells, thus driving invasion and metastasis. Furthermore, prescription of certain VGSC-inhibiting medications has been associated with reduced cancer incidence and improved survival in several observational studies. Thus, VGSC-inhibiting drugs already in clinical use may be ideal candidates for repurposing as possible anti-metastatic therapies. While these results are promising, further work is required to establish whether other VGSC inhibitors may afford superior metastasis suppression. Finally, increasing preclinical evidence suggests that several other ion channels are also key drivers of cancer hallmarks; thus, there are undoubtedly further opportunities to harness ion transport inhibition that should also be explored.

## Main text

Metastasis is the leading cause of cancer deaths; however, currently there are no agents in clinical use that specifically target this critically important hallmark of cancer. The development of therapies that can suppress invasion and prevent metastasis, ‘anti-metastatic drugs’, is an important area of unmet therapeutic need, and one which can have broad applicability across tumour types [1]. The new results of an open-label, multicentre randomised trial published in *J Clin Oncol* showed a significant disease-free survival (DFS) benefit for breast cancer patients receiving presurgical, peritumoral injection of lidocaine (HR 0.74; 95% CI 0.58–0.95; *p* = 0.017; n = 1583) [2]. Lidocaine is an amide local anaesthetic, which blocks voltage-gated sodium channels (VGSCs). VGSCs are transmembrane proteins which enable the passage of sodium ions in response to a change in voltage. They are expressed on electrically excitable cells, including neurons and cardiomyocytes, where they sustain rapid membrane depolarisation during action potential firing. As a result of this key biophysical function, VGSCs are important drug targets for excitability-related disorders, including epilepsy (e.g. using carbamazepine, lamotrigine, phenytoin, valproate), neuropathic pain (e.g. using lidocaine, carbamazepine), affective disorders (e.g. using tricyclic antidepressants) and cardiac arrhythmia (e.g. using flecainide, mexiletine, propafenone).

Transmembrane transport of sodium, which is elevated in malignant tumours, regulates acidosis, immune avoidance, invasion and metastasis [3, 4]. Persistent sodium influx drives glycolytic proton production and export (which metastatic cancer cells rely on to acidify and enzymatically digest the extracellular matrix), thus promoting early invasion [5]. A growing body of preclinical evidence highlights VGSCs as key protagonists in regulating this altered sodium influx in cancer cells, thus driving metastasis [5]. Moreover, VGSC inhibition in preclinical murine tumour models, using drugs including phenytoin, ranolazine and lidocaine, suppresses metastasis [3]. Given the wide array of well-tolerated VGSC-inhibiting drugs already in clinical use, such agents may therefore be ideal candidates for repurposing as possible anti-metastatic therapies. The common mechanism of action of these VGSC inhibitors is through directly occluding the ion-conducting pore. Such drugs preferentially bind to VGSCs in the so-called open or inactivated states; these states predominate in aberrantly hyperexcitable cells during seizure and/or arrhythmogenic activity, thus providing the basis for the drugs’ selectivity, and sparing electrical activity in normally functioning cells [6]. Importantly, VGSCs expressed on tumour cells also exist mainly in the open or inactivated states. These functional states permit persistent sodium influx and render tumour VGSCs highly susceptible to blockage by the same drugs [5].

Beyond the recent lidocaine study [2], there is some epidemiological data in support of targeting VGSCs in tumours. Although prescription of VGSC-inhibiting medications has been associated with reduced cancer incidence and recurrence in several observational studies, a recent cohort study in *BMJ Open* demonstrated that exposure to VGSC-inhibiting anticonvulsant medications is associated with shortened cancer-specific survival (HR 1.59, 95% CI 1.56–1.63, *p* < 0.001; n = 132,996) [7]. However, this negative association is likely due to confounding by indication because of epilepsy diagnosis. Interestingly, the same study showed that prescription of VGSC-inhibiting antiarrhythmic drugs which are not indicated for epilepsy, including the slow binding and persistent current blocking Vaughan Williams Class 1c and 1d agents, was associated with significantly improved survival (HR 0.75, 95% CI 0.64–0.88, *p* < 0.001 and HR 0.54, 95% CI 0.33–0.88, *p* = 0.01, respectively) [7]. These data therefore support the possibility that repurposing VGSC-inhibiting drugs as anti-cancer agents may result in therapeutic benefit by suppressing cellular migration and invasion and so preventing metastasis. This may be particularly important in the context of tumour resection, where surgery-induced inflammation and/or 'showering' of the surgical site with cancer cells is associated with increased tumour dissemination [8].

While these results are promising, further work is required to establish whether other widely used, safer and orally available VGSC inhibitors such as the antianginal ranolazine, may afford superior metastasis suppression, e.g. if treatment were commenced before surgery and for a defined period after surgery. Given that micrometastasis is likely have already occurred at diagnosis in many patients, systemic therapy may be more effective. In addition, potential limitations in the trial design should be addressed in future studies, e.g. patients receiving neoadjuvant chemotherapy should be included, patients should be stratified by margin positivity status, and timing of the intervention before vs. after extirpation should be explored. Furthermore, additional mechanisms that may contribute to the DFS improvement caused by lidocaine, e.g. Src family kinase inhibition 9, should be investigated.

The field is currently limited by the lack of a known biomarker of response for patient selection, which hampers development of preoperative window-of-opportunity studies that could provide early evidence of clinical activity, assess timing of administration and/or compare inhibitor type. Future work should address possible suitable biomarkers of response to VGSC inhibition, e.g. ctDNA [10]. Given the current lack of an ideal biomarker, any study may need to mirror the adjuvant ADD-ASPIRIN trial, which randomised patients with breast and colon cancer to placebo versus aspirin (ISRCTN: 74358648), based on preclinical and epidemiological studies, with an endpoint of invasive disease-free survival [11]. Finally, given the increasing preclinical evidence demonstrating that various other ion channels are also key drivers of cancer hallmarks, including cell cycle progression, proliferation, angiogenesis and apoptosis resistance [12], there are undoubtedly further opportunities to harness ion transport inhibition that should also be explored.

## Data Availability

Not applicable.
